# Cationic Peptide Exposure Enhances Pulsed-Electric-Field-Mediated Membrane Disruption

**DOI:** 10.1371/journal.pone.0092528

**Published:** 2014-03-26

**Authors:** Stephen M. Kennedy, Erik J. Aiken, Kaytlyn A. Beres, Adam R. Hahn, Samantha J. Kamin, Susan C. Hagness, John H. Booske, William L. Murphy

**Affiliations:** 1 School of Engineering and Applied Sciences, Harvard University, Cambridge, Massachusetts, United States of America; 2 Department of Electrical and Computer Engineering, University of Wisconsin, Madison, Wisconsin, United States of America; 3 Department of Biomedical Engineering, University of Wisconsin, Madison, Wisconsin, United States of America; 4 Department of Orthopedics and Rehabilitation, University of Wisconsin, Madison, Wisconsin, United States of America; National Research Council, Italy

## Abstract

**Background:**

The use of pulsed electric fields (PEFs) to irreversibly electroporate cells is a promising approach for destroying undesirable cells. This approach may gain enhanced applicability if the intensity of the PEF required to electrically disrupt cell membranes can be reduced via exposure to a molecular deliverable. This will be particularly impactful if that reduced PEF minimally influences cells that are not exposed to the deliverable. We hypothesized that the introduction of charged molecules to the cell surfaces would create regions of enhanced transmembrane electric potential in the vicinity of each charged molecule, thereby lowering the PEF intensity required to disrupt the plasma membranes. This study will therefore examine if exposure to cationic peptides can enhance a PEF’s ability to disrupt plasma membranes.

**Methodology/Principal Findings:**

We exposed leukemia cells to 40 μs PEFs in media containing varying concentrations of a cationic peptide, polyarginine. We observed the internalization of a membrane integrity indicator, propidium iodide (PI), in real time. Based on an individual cell’s PI fluorescence versus time signature, we were able to determine the relative degree of membrane disruption. When using 1–2 kV/cm, exposure to >50 μg/ml of polyarginine resulted in immediate and high levels of PI uptake, indicating severe membrane disruption, whereas in the absence of peptide, cells predominantly exhibited signatures indicative of no membrane disruption. Additionally, PI entered cells through the anode-facing membrane when exposed to cationic peptide, which was theoretically expected.

**Conclusions/Significance:**

Exposure to cationic peptides reduced the PEF intensity required to induce rapid and irreversible membrane disruption. Critically, peptide exposure reduced the PEF intensities required to elicit irreversible membrane disruption at normally sub-electroporation intensities. We believe that these cationic peptides, when coupled with current advancements in cell targeting techniques will be useful tools in applications where targeted destruction of unwanted cell populations is desired.

## Introduction

Cell membranes will develop aqueous pores in the presence of an electric field of appropriate duration and intensity [1]–[7]. This phenomenon is often referred to as electroporation [8]–[12]. Conventionally, an externally applied pulsed electric field (PEF) is used to generate the transmembrane electric potentials required for electropore development. If these electropores are transient, the cell membrane recovers and the cell can remain viable in a scenario referred to as transient electroporation (TEP). While open, membrane-impermeable entities gain entry into the cytosol, and thus TEP can be used to deliver entities such as peptides, full-length proteins, DNAs, RNAs [8], [13], [14], dyes, tracers, antibodies [15], metallic nanoparticles [16], and semi-conducting nanoparticles [17]. TEP can be detected through the inclusion of diagnostic membrane-impermeable molecules. If electropores remain protractedly open, the cell will not remain viable (i.e., irreversible electroporation (IEP)). Like TEP, IEP can be detected through the inclusion of diagnostic membrane-impermeable molecules. However, due to persistent membrane poration, IEP cells internalize relatively high quantities of these diagnostic molecules when compared to TEP cells. This has been demonstrated experimentally using both real-time imaging [18] and flow cytometry [19]. Because IEP is associated with irrevocable membrane damage, this form of electroporation has been used as a non-thermal ablation modality to destroy otherwise undesirable cells (e.g., bacteria and cells comprising tumors) [20]–[22]. For example, electroporation has been used to non-thermally ablate tumors subcutaneously implanted in mice [23]. A novel advantage to this approach when compared to other therapeutic strategies (e.g. chemotherapy, thermal ablation) is that electroporation only damages cell membranes, preserving tissue extracellular matrix components which are critical in post treatment tissue recovery [24].

We and others have hypothesized that electric potentials on the order of that required to achieve electroporation may be achieved through the co-localization of charged macromolecules and the plasma membrane. Binder and Lindbolm [25] proposed an “electroporation-like” mechanism for the internalization of penetratin (a cationic peptide), and demonstrated that its internalization is ATP- and temperature-independent. This suggests that its internalization is non-endocytic. They also demonstrated that penetratin internalization was charge-dependent, arguing that its internalization is based on an electroporative mechanism. Wadia and Dowdy [26] found that the internalization efficiency of several cell penetrating peptides (CPPs) is correlated to the number of arginines (cationic residues) in the CPP’s amino acid sequence. Wender *et al* [27] demonstrated that replacing the arginines in the internalization sub-domain of TAT (a recognized CPP) with neutral residues reduces internalization efficiency by 70 to 90 percent. The cationic CPP perforin was shown to trigger the membrane repair response in HeLa and CHO-K1 cells [28], suggesting that perforin exposure resulted in plasma membrane disruption. Lastly, Miteva *et al* [29] calculated that the electrostatic potential created by the co-localization of NK-lysin and the cationic peptide sub-domain of NK-lysin (LFS**R**MI**KK**CLG**R**L, cationic residues are indicated by bold type) were above 0.2 and as high as 0.4 V. Remarkably, each of these studies has demonstrated electroporation-like effects of polycationic molecules in the absence of an externally applied electric field.

**Figure 1:**
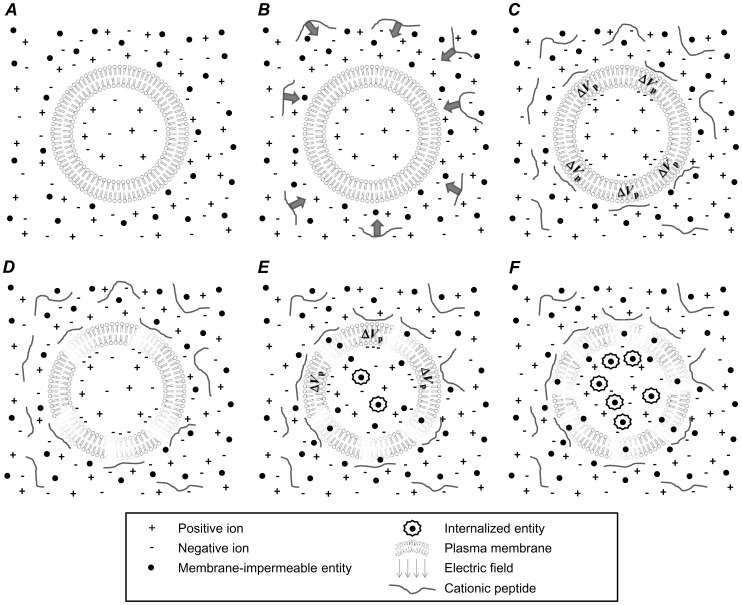
Cationic peptide exposure can eventually lead to prolonged membrane disruption through electrostatic peptide-membrane interaction. A cell rests in an ionic media with membrane-impermeable entities (***A***) and cationic peptide is introduced into that media (***B***). Cationic peptides will electrostatically collect about the negatively charged plasma membrane (***C***) and increase the electrostatic potential across the membrane in the vicinity of the peptide (*V*_p_). ***D***–***E***: These transmembrane voltages may be sufficient for electroporation and disrupt the membrane in a manner leading to internalization of normally membrane-impermeable entities. ***E***–***F***: Particularly at higher cationic peptide concentrations, the media serves as a reservoir for additional peptides to co-localize with the plasma membrane, causing prolonged membrane permeability.

The findings described above strongly suggest that cationic peptides are capable of electrostatically interacting with the plasma membrane and creating localized regions where the membrane’s electrostatic potential is enhanced in the vicinity of the charged peptide. This concept is illustrated in Fig. 1. When added to cell media (Fig. 1, ***A***), cationic peptides will be electrostatically pulled towards the cell’s plasma membrane due to the negatively charged phosphatidylserine (PS) phospholipids contained in plasma membrane’s intracellular lipid layer (Fig. 1, ***B***). This use of positively charged molecules to electrostatically home to negatively charged cell surfaces is exploited in numerous clinical and research settings, notably in gene delivery, where DNA molecules are complexed with polycations [30], [31]. This complexation is done in order to (i) condense the genetic materials to sizes that are capable of being internalized, and critically, (ii) endow the DNA-polycation complexes with net positive charges, allowing them to electrostatically collect about anionic cell surfaces [32]. Thus, increased positive charge results in enhanced co-localization to cell surfaces, increased DNA uptake, and improved transfection [33]–[35]. While (i) shielding effects from mobile counter-ions in the cell media and (ii) the intracellularly facing location of negatively charged PS lipids may reduce the capacity of cationic molecules to be electrostatically pulled to the surface of cells, there is a wealth of evidence demonstrating that cationic peptides [25]–[27] and cationic DNA complexes [30]–[35] do in fact co-localize with the surface of cells because of electrostatic affinity. In fact, electrostatic interactions between molecules and structures (despite being in ionic solutions containing counter-ions) are fundamentally exploited by nature in a wide range of biological processes. Thus, after addition, cationic peptides will collect about the plasma membrane. Upon co-localization, they can create regions where the cell membrane’s electrostatic potential is enhanced proximally near individual peptides by attracting counter ions to the inner cytosolic membrane surface (Fig. 1, ***C***, *V*_p_). If these locally enhanced electrostatic potentials are sufficient to induce electroporation, the cell membrane will be disrupted (Fig. 1, ***D***), leading to the internalization of extracellular membrane-impermeable entities (Fig. 1, ***E***). This membrane disruption should persist, especially at high cationic peptide concentrations, due to there being an ample reservoir of peptides in the media continually allowing for membrane disruption and internalization of membrane-impermeable entities (Fig. 1, ***E***–***F***).

**Figure 2:**
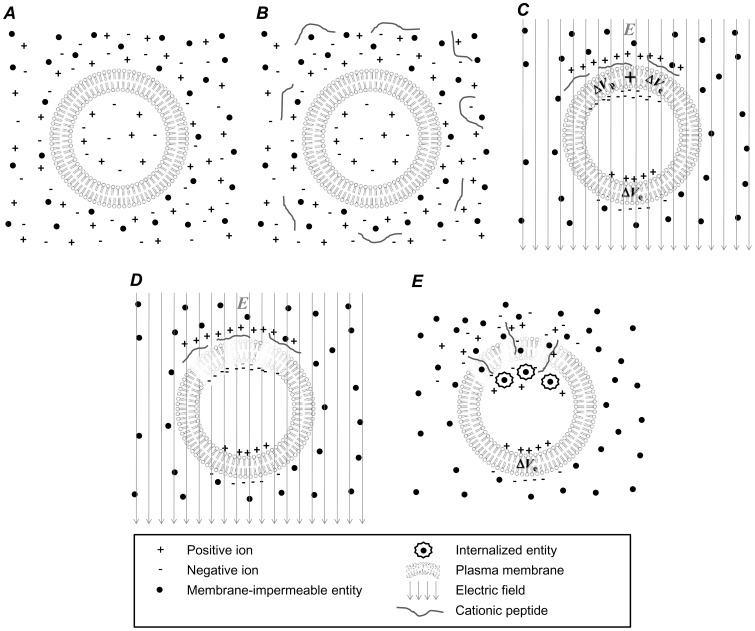
When used in concert with an externally applied PEF, cationic peptides can be used to enhance membrane disruption at lower PEF intensities at the anode-facing cell membrane. When a cell rests in an ionic medium containing membrane impermeable entities (***A***) and cationic peptides are added to that medium (***B***), a PEF can be used to accumulate those cationic peptides about the anode-facing membrane (***C***) where the anode-facing membrane can experience a local increases in electrostatic potential near individual peptides (*V*_p_) plus enhanced electrostatic potential due to the ion relocation due to the applied field (*V*_e_). These local enhancements in transmembrane electrostatic potentials can result in anodally preferenced electroporation at lower PEF intensities (***D***) and subsequent internalization of normally membrane-impermeable entities through the anode-facing membrane (***E***).

It is possible that cationic peptide exposure may also be used to lower the PEF intensities required to disrupt the plasma membrane. This concept is illustrated in Fig. 2. Again, a cell rests in an electrolytic medium (Fig. 2, ***A***) and cationic peptides are introduced to the medium (Fig. 2, ***B***). If a PEF is applied prior to significant electrostatically induced peptide accumulation about the membrane, peptides will electrophoretically accumulate at the anode-facing membrane surface (Fig. 2, ***C***). We hypothesized that this PEF-induced anodic peptide accumulation can result in peptide-proximal enhancements in transmembrane electric potential (Fig. 2, ***C***, *V*_p_), rendering the membrane more susceptible to electroporation in the vicinity of those peptides. That is, the electric potential near accumulated peptides will be increased by a combination of peptide co-localization and PEF-induced ion accumulation (Fig. 2, ***C***, *V*_p_+*V*_e_). As a result, it may be possible to disrupt the anodic membrane at lower PEF intensities (Fig. 2, ***D***), resulting in molecular uptake preferentially through the anodic membrane (Fig. 2, ***E***). In this study, we aim to determine if cationic peptide exposure can contribute to membrane disruption and enhance the ability of an externally applied PEF to incur irreversible disruption of plasma membranes.

## Materials and Methods

### Experimental Setup

**Figure 3:**
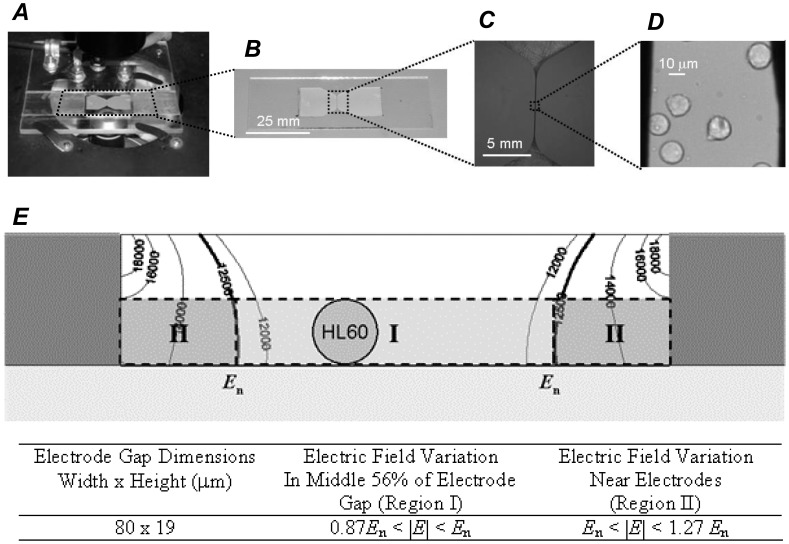
A microcuvette with a microchannel was used to expose cells to PEFs while imaging them over time under fluorescence and bright-field microscopy. ***A***: The microcuvette was designed to be placed on the platform of an inverted microscope. ***B***–***D***: The microcuvette has an 80 mm microchannel where cells can be exposed to PEFs between two parallel electrodes and monitored under microscopy. ***E***: FEM analysis results showing field heterogeneity within the microcuvette’s microchannel when the electrodes are excited with a nominal electric field value of *E*_n_ = 12.5 kV/cm (as calculated by the excitation voltage divided by the electrode gap). HL60 s resting in region I would be exposed to 0.87*E*_n_ to *E*_n_ while cells resting in region II would be exposed to *E*_n_ to 1.27*E*_n_. However, because of the steep field gradient in region II, only cells in region I were analyzed for this study. Thus, for a given experiment, the range in PEF intensities to which a given cell was exposed was known. Part ***E*** was adapted from Kennedy et al. [18] with authors’ permission.

PEFs were applied using a BTX Model ECM 830 Electro Square Porator (Harvard Apparatus, Holliston, MA, USA). The output of the BTX Electroporator was connected to a platform that held a device previously developed for microscopically observing cells during electroporation–the *microcuvette* [18]. Fig. 3 provides images of this platform (Fig. 3, ***A***) and the microcuvette under increasing levels of magnification (Fig. 3, ***B***–***D***). Cells reside in the microcuvette’s micro-channel (19 μm high and 80 μm wide) during experimentation. This pulsing system provided monopolar, square pulses with voltages up to 3 kV and pulse widths as narrow as 10 μs, though 40 μs PEFs were used these studies.

The microcuvette’s platform was fixed to the platform of a Nikon Eclipse TE200 microscope (Nikon USA, Melville, NY, USA) equipped with a Hamamatsu C4742-95 Digital charge-coupled device (CCD) camera (Hamamatsu Photonics, Bridgewater, NJ, USA). Propidium iodide (PI) fluorescence was used as an electroporation indicator. PI is membrane impermeable and will traverse cellular membranes only when membrane integrity has been compromised. Once PI molecules have traversed the plasma membrane they will bind to available intracellular nucleic acids, dramatically increasing their fluorescence capacity. PI fluorescence was monitored via fluorescence microscopy. Filters were selected to excite nucleic acid-bound PI at 535 nm using a Lambda DG-4 excitation lamp/high speed wavelength switcher (Sutter Instrument, Novato, CA, USA) and monitor its fluorescence at 617 nm. Control and timing of the excitation lamp and CCD camera were provided by PC software (Prairie Technologies Inc., Madison, WI, USA). Image analysis was performed using MATLAB (The MathWorks, Natick, MA, USA). CCD camera exposure time and gain settings were set, establishing detection sensitivities appropriate for analyzing electroporative delivery dynamics corresponding to a wide range in PI fluorescence. It was determined that the estimated minimum detectible amount of PI fluorescence for this experimental setup is between 10.6 and 23.2 million PI molecules per cell while the estimated maximum is approximately 500 million PI molecules per cell [18]. We were also able to estimate the number of internalized PI molecules per cell based on individual cellular PI fluorescence by applying a calibration curve as described in Kennedy *et al*. [18]. The microcuvette’s electrode microchannel was 80 um wide and 19 um high. Thus field inhomogeneity was expected and confirmed with a finite element electric field heterogeneity analysis of the microcuvette’s microchannel [18]. In these studies, the proximity of an individual cell to an electrode was used to estimate the electric field intensity to which an individual cell was exposed (Fig. 3, ***E***). Because cells residing near either electrode experienced not only heightened electric fields, but a significant field gradient over the length-scale of a cell (Fig. 3, ***E***, Region II), kinetic PI uptake versus time data was not collected from these cells. For cells residing in the middle 56% of the microchannel, the PEF intensities exposed to those cells were determined to range from as high as the nominal field intensity (*E*_n_, as calculated by the electrode excitation voltage divided by the separation between electrodes) to as low as 0.87*E*_n_.

### Cell Culture Maintenance and Experimental Protocols

HL60 human promyelocytic leukemia cells (American Type Culture Collection, Manassas, VA, USA) were chosen for all experiments. HL60 s were chosen here as they have mostly simple spherical geometries. This allows any analyses regarding the location of membrane disruption visually discernable and less subject to morphological complexities. HL60 s were cultured in RPMI-1640 containing 2% glutamine supplemented with 10% fetal bovine serum (FBS) and 2% penicillin and streptomycin at 37°C and 5% CO_2_. Prior to experimentation, cells were re-suspended in HBSS without Ca^2+^ and Mg^2+^ and without phenol red (conductivity of 1.42 S/m) at approximately 1 million cells/ml. PI (Fisher Scientific International, Pittsburgh, PA, USA) was added to the cell/HBSS mixture to form a PI concentration of 60 μM.

Prior to experimentation, microcuvettes were treated with a poly-L-lysine adhesion coating by adding 10 μL of poly-L-lysine (Newcomer Supply, Middleton, WI, USA) on top of the microcuvette’s microchannel. Microcuvettes were then thoroughly rinsed in a petri dish containing 20 mL of deionized water in order to remove un-absorbed poly-L-lysine. The microcuvettes where then removed from the deionized water and allowed to dry in the biosafety cabinet for 1 hour. They were then transferred to the electroporator/microscope platform. Note that poly-L-lysine is itself a cationic peptide and can therefore (i) potentially have a detrimental influence over cell viability over the course of these experiments and (ii) could interfere with our ability to observe the influence of soluble polyarginine on reducing PEF intensities required to cause membrane disruption. Control experiments were therefore conducted with HL60 s resting on poly-L-lysine-treated substrates where no polyarginine was provided and no PEF was administered to see if this adhesion layer influenced membrane integrity during experimental timeframes. Regarding potential contributions of cationic poly-L-lysines in enhancing PEF-mediated membrane disruption, we believe poly-L-lysine does not play a role here. Because the surfaces of the cells that are exposed to the poly-L-lysine substrate are orthogonal to the PEF, the poly-L-lysine surface should have little impact on experimental outcomes. Nonetheless, experiments where cells were and were not exposed to polyarginine both were conducted on poly-L-lysine treated substrates. Thus, analyses of the experimental outcomes were made based on differences between the two cases. After poly-L-lysine treatment, a Teflon washer was placed on top of the microcuvette’s microchannel and used to contain liquid during experimentation. A 10 μL volume of cell/HBSS/PI was then injected into this well and a cover slip was immediately placed on top of the washer to limit evaporation during experimentation. Then, 5 minutes were provided before beginning the experimental time course to allow cells to settle in the microcuvette’s microchannel.

**Figure 4:**
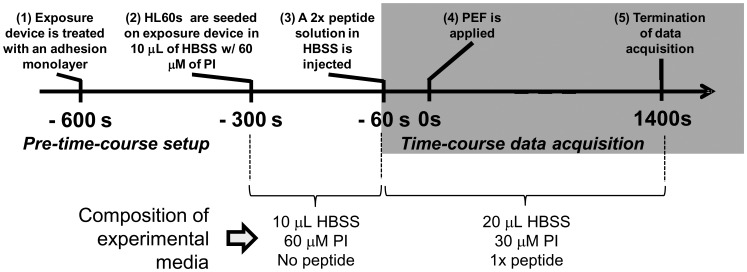
A timeline summarizing the experimental protocol. The microcuvette was treated with an adhesion peptide so that cells would settle into the microchannel and remain immobilized during experimentation. Cells and PI were added to the microcuvette 300 seconds prior to PEF exposure and allowed to settle into the microchannel. If peptides were used, at 60 seconds before PEF exposure, they were added at twice their desired concentration so that, when combined with the cell/PI mixture in a 1∶1 ratio, they would dilute into the desired experimental concentration. Also at 60 seconds prior to PEF exposure, fluorescence imaging of the cells began (1 image every 10 seconds). At time 0, a PEF was administered and cells were imaged for 1400 seconds.

It was previously determined that 300–400 seconds were required after the addition of concentrated cationic peptides (at 250 μg/ml of polyarginine) to begin to have an effect on HL60 cells’ membrane integrity. We therefore decided that 60 seconds would be provided between the addition of cationic peptide and PEF application. This would allow enough time for the cationic peptides to diffuse and distribute in the experimental medium while not yet affecting cell membranes. Thus, 60 seconds prior to PEF application (at *t* = −60 s), 10 μL of a 2× peptide/HBSS solution was injected into the microchannel (2× indicates that the peptide concentration is twice the concentration that is desired for a given experiment). This resulted in an experimental solution that contains 30 μM PI in HBSS and 1× of the selected peptide. Fluorescence microscopy image acquisition was initiated immediately after peptide injection (1 fluorescent image every 10 seconds). At time *t* = 0 s, a 40 μs PEF was applied and image acquisition continued for 1400 seconds. A summary timeline of the experimental protocol is provided in Fig. 4.

Peptide concentration type and concentration varied based on the experiment. The peptides used in these experiments included polyarginine which ranged from 15 to 70 kDa and the neutral polyasparagine (Sigma-Aldrich, St. Louis, MO, USA). Addition of 250 μg/mL polyarginine and polyasparagine (the highest concentrations used in these studies) were verified to have no detectible impact on the pH of experimental solutions. Additionally, addition of 250 μg/mL polyarginine had no detectible impact on HBSS conductivity (both were measured at 1.42 S/m). Calculations determined that this polyarginine addition results in a 9 μM increase in osmotic concentration and a 0.000387 S/m increase in electrical conductivity which is consistent with our inability to detect conductivity differences when measured. While these changes are not negligible, we do believe they will not have an impact on experimental outcome, particularly since we are using relatively high conductivity experimental media (1.42 S/m). Ivorra et al. [36] showed that changes in medium conductivity can have a significant impact on electroporative outcome; however, these differences manifested themselves most when using low-conductivity media. Specifically, electroporative responses varied greatly based on changes in medium conductivity at conductivities lower than 0.1 S/m and did not vary at conductivities higher than that. This was experimentally corroborated in a number of different cell types using a variety of metrics [37]–[39].

To better understand how PI versus time curves related to electropore closure/persistence and general severity, we conducted experiments where cells were exposed to 40 μs PEFs with PI present and where a secondary membrane integrity indicator, trypan blue (TB), was added 30 minutes later. Before adding TB, 30 minutes was allowed for transient electropores to seal, thereby excluding TB but internalizing some amount of PI. Irreversibly electroporated cells would expectedly include TB and internalize large quantities of PI. Non-electroporated cells would expectedly internalize neither PI nor TB. After adding TB, cells were transferred to a standard microscope slide and examined under bright-field and fluorescence microscopy to assess TB and PI internalization, respectively. In order to minimize the effects of evaporation over >30 minute time periods, standard 2 mm electroporation cuvettes were used in these experiments, allowing for the use of larger volumes.

### Defining and Categorizing PI Uptake Signatures

**Figure 5:**
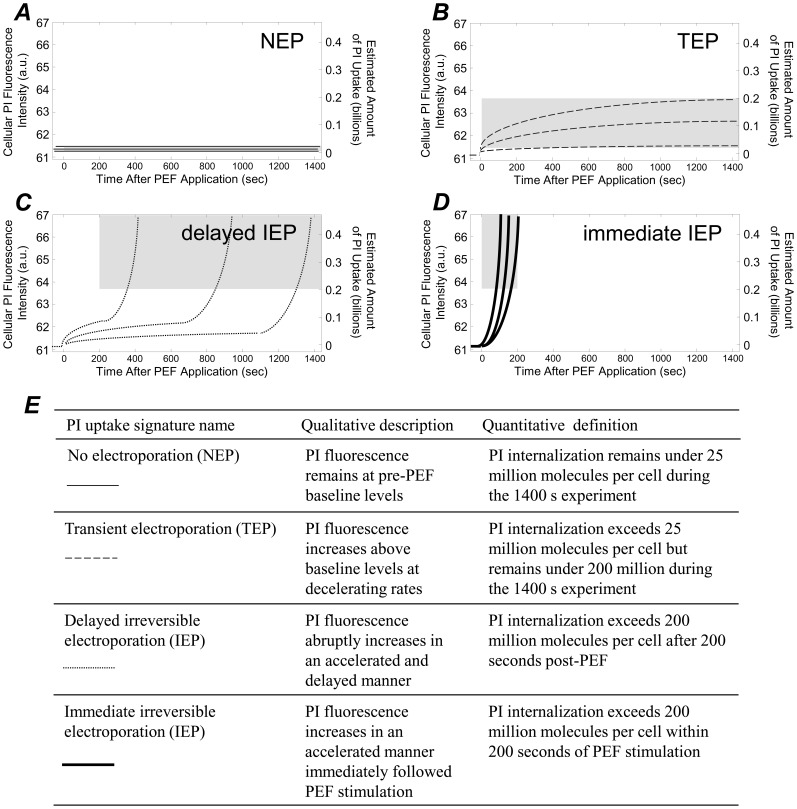
PI uptake versus time signatures from every experiment from each cell fell into one of four categories, as defined here. ***A***: The NEP signature is assigned to cells that remain at baseline PI fluorescence levels of <25 million molecules per cell during the 1400 second experimental time course. ***B***: The TEP signature is assigned to cells that internalize between 25 and 200 million PI molecules per cell during the 1400 second time course. ***C***: The delayed IEP signature is assigned when cells internalize more than 200 million PI molecules per cell between 200 and 1400 seconds after PEF stimulation. ***D***: The immediate IEP signature is assigned when cells internalize more than 200 million PI molecules within the first 200 seconds after PEF exposure. ***E***: A table summarizing the names, qualities, and quantitative definitions of each signature. In ***A***–***D***, grey regions on plots are provided to indicate the widows defining the uptake signature in terms of PI uptake amount and timeframes.

The PI versus time signatures captured in all our experiments always fell into one of four categories. These categories are defined here with the aid of Fig. 5. Elsewhere, these uptake signatures have been used to describe electroporative response, with particular emphasis on how the kinetics of such signatures are indicators of the relative severity of membrane disruption [18], [40]. HL60 s sometimes exhibited PI fluorescence levels that remained at baseline levels during the entire course of the 1400 s experiment (Fig. 5, ***A***). These cells internalized undetectable quantities of PI (i.e., fewer than 25 million PI molecules per cell). This baseline PI uptake signature is consistent with NEP. Therefore, we will refer cells whose PI uptake signature remains under 25 million PI molecules per cell over the entire 1400-second experiment as NEP-exhibiting cells. HL60 s also exhibited PI uptake signatures that were consistent with TEP. These cells internalize detectible quantities of PI but fluorescence levels plateaued at relatively low intensities (Fig. 5, ***B***). This plateauing is consistent with a cessation of PI internalization due to membrane recovery and the relatively low amounts of internalization are also an excellent indicator of TEP [18], [19]. Thus, we will define cells whose PI uptake plateaus between 25 and 200 million PI molecules per cell during the 1400-second experiment as TEP-exhibiting cells (Fig. 5, ***B***). The two remaining PI uptake signatures are consistent with irreversible membrane disruption. PEF-induced irreversible membrane disruption corresponds to high levels of molecular uptake through relatively numerous and large membrane pores [19] at accelerating rates [18], [40]. This describes the two remaining PI uptake signatures which are both characterized by high levels of PI fluorescence and accelerating rates of PI uptake (Fig. 5, ***C*** and ***D***). When this accelerating, high-level PI uptake follows immediately after PEF stimulation (Fig. 5, ***D***), this is consistent with the development of numerous and large membrane pores during PEF stimulation. Thus, when a cell internalizes large quantities of PI (i.e., >200 million PI molecules per cell) quickly after PEF stimulation (i.e., within 200 seconds post-PEF), we will refer to that cell as an “immediate IEP”-exhibiting cell. When this accelerating, high-level PI uptake manifests itself in a delayed manner (Fig. 5, ***C***), we will refer to this as “delayed IEP.” Delayed IEP-exhibiting cells internalize greater than 200 million PI molecules per cell but exceed this amount of between 200 and 1400 seconds after PEF stimulation. A summary of how these PI uptake signatures are quantitatively defined and labeled on plots are included in the table in Fig. 5, ***E***.

### Statistical Analysis

When error bars are used, data are expressed as means ± standard deviations from data collected across multiple individual experiments (N). These data were analyzed by one-way Analysis of Variance (ANOVA) to determine statistical significance using KaleidaGraph, version 4.1 (Synergy Software, Reading, PA). When significant *F*-ratios were obtained, post hoc multiple comparisons were performed using Tukey’s protected least-significance difference tests to determine whether specific differences had occurred between groups.

## Results and Discussion

### Progression of PI Uptake Signatures at Varying PEF Intensities with no Peptide Present

**Figure 6:**
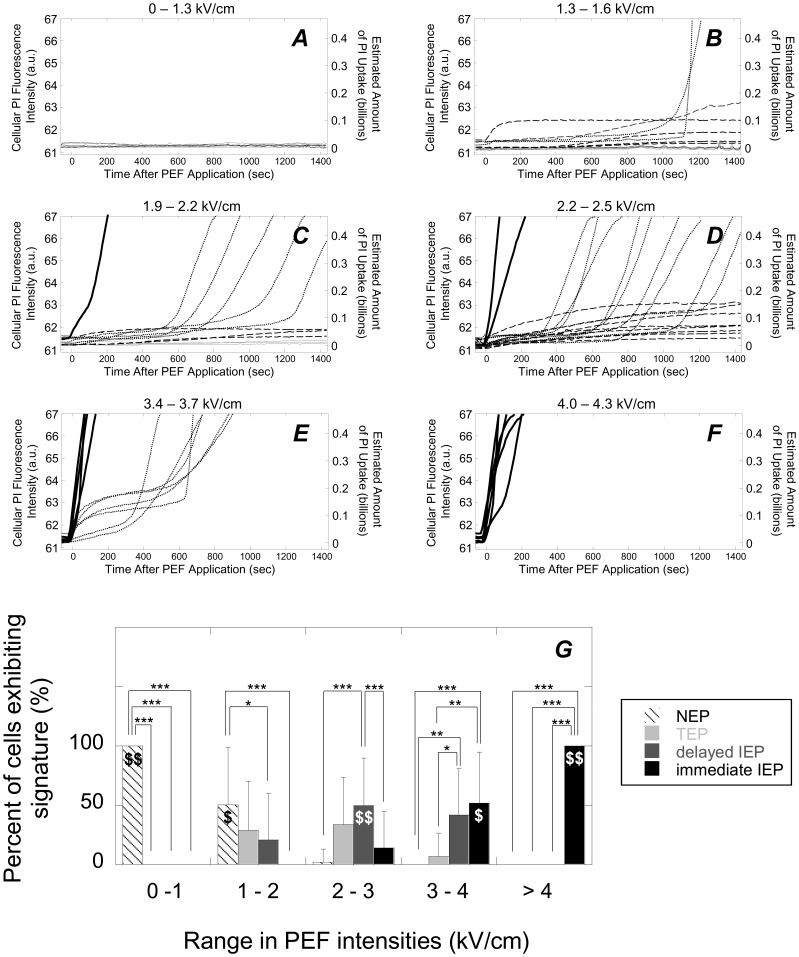
When no peptides were used, use of progressively more intense PEFs resulted in PI uptake signatures that were indicative of progressively higher degrees of membrane disruption. ***A–F***: Representative PI uptake versus time curves at the indicated ranges in PEF intensities highlighting general trends and emergence and disappearance of different uptake signatures. Light solid lines, dashed lines, dotted lines, and bold solid lines, indicate NEP, TEP, delayed IEP, and immediate IEP signatures, respectively. Under 1.3 kV/cm, cells exclusively exhibited NEP signatures (***A***) and began exhibiting TEP and delayed IEP signatures above 1.3 kV/cm (***B***). Above 1.9 kV/cm, immediate IEP signatures were first observed (***C***). Above 2.2 kV/cm, NEP signatures were no longer observed (***D***) and above 3.4 kV/cm, the TEP signatures were no longer observed (***E***). Finally, above 4.0 kV/cm, the immediate IEP signature was exclusively observed (***F***). ***G***: A plot summarizing the data for all 80 experiments involving 269 cells. Data are represented as means and standard deviations. *, **, and *** indicate statistical significances of *p*<0.05, 0.01, and 0.001, respectively, when comparing individual signatures to other individual signatures. $ and $$ indicate statistical significance of *p*<0.01 and 0.001, respectively, when comparing an individual signature to all other signatures combined within that PEF range. The number of independent experiments represented in 0–1, 1–2, 2–3, and 3–4 kV/cm bins are *N* = 3, 29, 30, 15, and 3, respectively. The total number of cells examined in 0–1, 1–2, 2–3, and 3–4 kV/cm bins were 28, 83, 106, 45, and 7, respectively.

In the absence of peptide, cells generally exhibited uptake signatures that were indicative of higher degrees of membrane disruption when cells were exposed to PEFs of increasing intensity. Figure 6, ***A***–***F*** shows PI uptake versus time kinetic data that is representative of observed trends and Fig. 6, ***G*** shows data representative of all 65 separate experiments involving 269 individual cells conducted in the absence of peptide. At PEFs under 1.3 kV/cm, all cells did not internalize detectable quantities of PI (Fig. 6, ***A***) but did internalize PI at 1.3–1.6 kV/cm (Fig. 6, ***B***, cells internalized >25 million PI molecules). That is, at 1.3 kV/cm cells transition from exhibiting NEP signatures (Fig. 6, ***A***, indicated by a light solid curve) to TEP and delayed IEP signatures (Fig. 6, ***B***, indicated by dashed and dotted curves, respectively), though NEP signatures were still detected (Fig. 6, ***B***, indicated by light solid curves). When using PEFs between 1.9–2.2 kV/cm, some immediate IEP signatures were observed and higher proportions of cells exhibited the delayed IEP signature (Fig. 6, ***C***). When using PEFs between 2.2–2.5 kV/cm, cells exhibiting the NEP signature were no longer detected and more cells exhibited the immediate IEP signature (Fig. 6, ***D***). When using 3.4–3.7 kV/cm, cells only exhibited the delayed and immediate IEP signatures (Fig. 6, ***E***) and above 4.0 kV/cm, cells only exhibited the immediate IEP signature (Fig. 6, ***F***).

These data are consistent with previously reported findings. The PEF intensity, where cells transition from not being electroporated to being electroporated (1.3 kV/cm), is consistent with another report where flow cytometry was used to quantify PI uptake in mouse myeloma cells in response to a 40 μs PEF in media containing 25 μg/mL PI (20 μg/mL is used in our studies). Müller and coworkers [40] found that PI uptake was not detectible at PEFs ≤1.0 kV/cm but were at 1.5 kV/cm. Furthermore, the amounts of PI uptake detected in TEP cells here (Fig. 6, ***B***–***D***, dashed lines indicating PI uptake in the low hundreds of millions of PI molecules per cell) are similar to those detected by Müller and coworkers (0.3 fmol/cell or ∼ 200 million molecules per cell) [19]. While Müller et al.’s use of flow cytometry provided excellent statistics and showed that more intense PEFs result in higher degrees of PI internalization, these kinetic data provide more insight with regard to the dynamics of membrane disruption. For instance, the timescales associated with PI uptake in TEP cells were consistent with other reports. We observed plateauing of the PI fluorescence over the course of hundreds of seconds (Fig. 6, ***B***–***D***, dashed curves). Similar transient electropore lifetimes have been experimentally corroborated in a number of different cell types using several different experimental metrics [18], [41]–[43], as well as theoretically [44].

At different ranges of PEF intensities, membrane responses–as defined by the different uptake signatures–tend to statistically dominate. For instance, at low PEF intensities (0 to 1 kV/cm), NEP statistically dominates (Fig. 6, ***G***, 0–1 kV/cm bin, comparing NEP to all other signatures). Again, this is consistent with flow cytometry data collected in a previous study [19]. In the range of 1–2 kV/cm, the NEP signature is still statistically more well represented than both the IEP signatures but not statistically different than the TEP signature (Fig. 6, ***G***, 1–2 kV/cm bin). In the range of 2–3 kV/cm the delayed IEP becomes statistically more prevalent than TEP and immediate IEP (Fig. 6, ***G***, 2–3 kV/cm bin, comparing delayed IEP to TEP and immediate IEP), but not statistically more prevalent than TEP. In the range of 3–4 kV/cm, the signatures that are indicative of irreversible membrane disruption (i.e., delayed/immediate IEP) are both statistically more prevalent than NEP and TEP signatures (Fig. 6, ***G***, 3–4 kV/cm bin). Finally, above 4 kV/cm, the immediate IEP signature statistically dominates all other signatures and was exclusively exhibited. Thus is appears that from 0 to 1 kV/cm NEP dominates and is exclusively observed, from 1 to 2 kV/cm NEP still dominates, from 2 to 3 kV/cm delayed IEP dominates, from 3 to 4 kV/cm immediate IEP dominates, and above 4 kV/cm immediate IEP dominates and is exclusively observed. This is given statistical weight when each of these signatures is compared to all the other signatures in that PEF range combined. That is, NEP is statistically more prevalent from 0 to 1 kV/cm than all other signatures combined (Fig. 6, G, $$ indicating *p*<0.001). Similar statistics were carried out within each PEF range: (i) NEP with *p*<0.01 from 1 to 2 kV/cm, (ii) delayed IEP with *p*<0.001 from 2 to 3 kV/cm, (iii) immediate IEP with *p*<0.01 from 3 to 4 kV/cm, and (iv) immediate IEP with *p*<0.001 above 4 kV/cm.

### Relationship between PI uptake Signature and Relative Degree of Membrane Disruption

**Figure 7:**
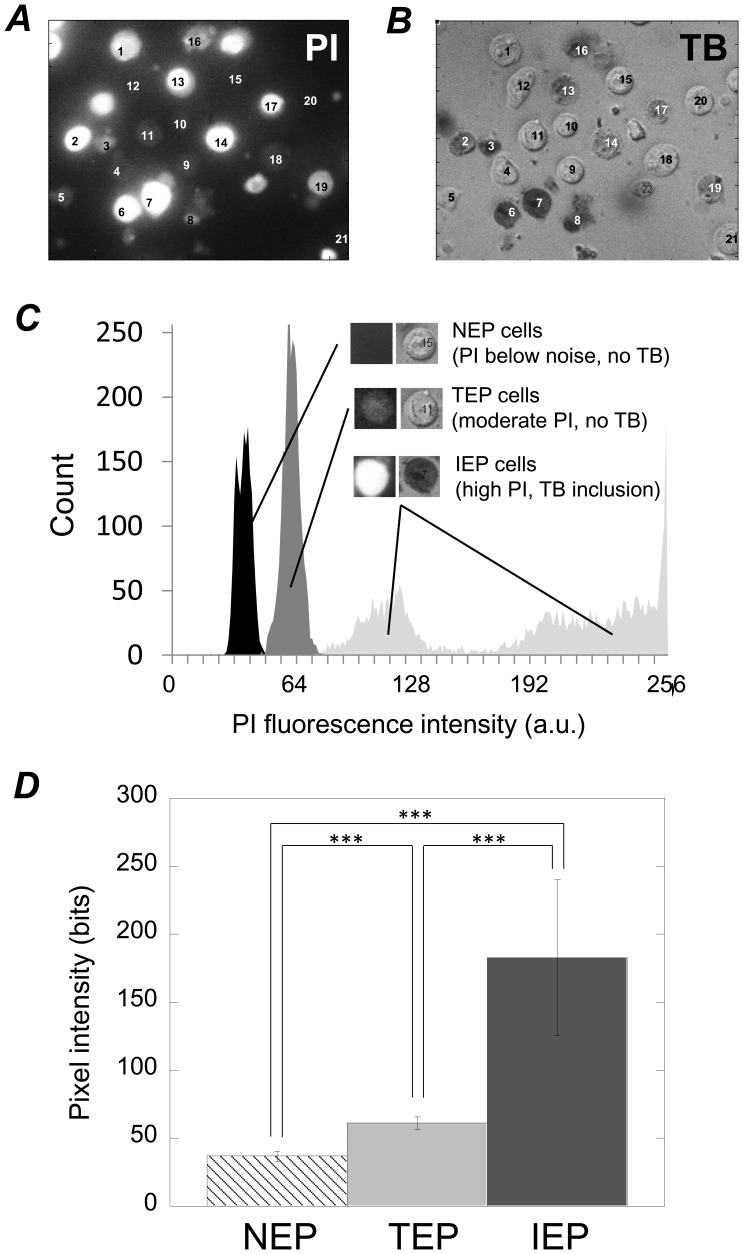
Different PI uptake signatures were associated with different degrees of membrane disruption. Representative images of PI fluorescence at the end of a 1400-second experiment (***A***) and TB inclusion/exclusion for those same cells immediately subsequent to TB addition 30 minutes after PEF administration (***B***). These images highlight the correlation between the relative intensity of PI fluorescence and TB inclusion verses exclusion. That is, NEP cells (cells 12, 15, 20, and 21) do not fluoresce at detectible levels and do not internalize TB. TEP cells (1, 4, 5, 9, 10, 11, and 18) moderately fluoresce and do not internalize TB. IEP cells (2, 3, 6, 7, 8, 13, 14, 16, 17, and 19) fluoresce brightly and do internalize TB. ***C***: NEP, TEP, and IEP cells fluoresce at discernibly different fluorescence levels as shown by a fluorescence pixel intensity histogram. ***D***: These fluorescence values differ in a statistically significant manner. In ***C***–***D***, fluorescence data was obtained from 41 separate experiments involving the use of PEFs ranging in intensity from 0 to 6.7 kV/cm, wherein 974 individual cells were analyzed. In ***D***, *** indicates statistical significance of *p*<0.001. The data represent pixel counts of N = 1765, 2701, and 4022 for the NEP, TEP, and IEP cases, respectively.

Based on these findings, we can determine how different PI uptake signatures are indicative of different degrees of membrane disruption. NEP-exhibiting cells predominantly resulted from lower PEF intensities (Fig. 6, ***G***), exhibited baseline fluorescence levels over time (Fig. 6, ***A***, ***B***, and ***C***), exhibited PI fluorescence levels under the detection threshold (Fig. 7, ***A***, cells 12, 15, 20, and 21), did not internalize TB when added 30 minutes after PEF administration (Fig. 7, ***B***, cells 12, 15, 20, and 21), and fluoresced at levels that were statistically lower than cells exhibiting the TEP and IEP signatures (Fig. 7, ***C*** and ***D***). Taken altogether, the NEP signature is consistent with there being no or undetectable degrees of membrane disruption. Cells exhibiting the TEP signature internalized PI in a decelerating manner (Fig. 6, ***B***, ***C***, and ***D***, dashed curves with negative concavities), only internalized under 200 million PI molecules per cell (also Fig. 6, ***B***, ***C***, and ***D***), and fluoresced at levels that were statistically higher than NEP-exhibiting cells but lower than IEP-exhibiting cells (Fig. 7, ***C*** and ***D***). The decelerating nature of PI uptake in these cells combined with the relatively moderate amounts of PI uptake (∼ 200 million molecules per cell (which is similar to other transient electroporation reports [19]) both suggest that the membrane permitted PI uptake initially but PI permeability decreased over time, eventually ceasing. This indicates that membrane disruptions were temporary. Additionally, while TEP-exhibiting cells did internalize moderate amounts of PI (Fig. 7, ***A***, cells 1, 4, 5, 9, 10, 11, and 18), they did not internalize TB when it was added 30 minutes after PEF administration (Fig. 7, ***B***, cells 1, 4, 5, 9, 10, 11, and 18), thus further indicating that TEP cells recover from membrane disruption. Added to the fact that TEP signatures were generated at relatively moderate PEF intensities (Fig. 6, ***G***, between 1 and 4 kV/cm and do not exist above 4 kV), the TEP signature is consistent with temporarily and relatively mild membrane disruption. Cells exhibiting the IEP signatures (both the delayed and immediate manifestations) internalized PI in an accelerating manner (Fig. 6, ***B***–***F***, dotted and solid bold curves with positive concavities), internalized over 200 million PI molecules per cell, and fluoresced at levels that were statistically greater than both NEP and TEP cells (Fig. 7, ***D***). This suggests that IEP signatures represent higher degrees of membrane disruption when compared to the NEP and TEP signatures. Additionally, IEP cells internalized TB when it was added 30 minutes post PEF administration (Fig. 7, ***B***, cells 2, 3, 6, 7, 8, 13, 14, 16, 17, and 19), further indicating that IEP signatures represent prolonged membrane disruption. Added to the fact that the IEP signatures occurred at relatively high PEF intensities (Fig. 6, ***G***), the IEP signatures are consistent with relatively severe and irreversible membrane disruption.

We believe that the immediate IEP signature indicates a more severe response than the delayed IEP signature. First, the immediate IEP signature was generally manifested at higher PEF intensities than the delayed IEP signature. For instance, at 2–3 kV/cm the delayed IEP signature was statistically more prevalent than the immediate IEP signature (Fig. 6, ***G***). At 3–4 kV/cm, the prevalence of the immediate IEP signature was statistically similar to that of the delayed IEP signature, and above 4 kV/cm the immediate IEP dominated (again, Fig. 6, ***G***). Additionally, the immediate IEP signature was associated with higher levels of PI internalization at earlier time points (Fig. 6, ***B***–***F***, comparing solid bold curves to dotted curves). This indicates that membranes were disrupted more extensively during the duration of PEF exposure, facilitating high quantities of PI internalization at early time points. Taken together, it seems the immediate IEP signature indicated more severe membrane disruption as generated as a result of PEF exposure. The dynamical differences in membrane responses that lead to the delayed versus immediate manifestations of the IEP signatures are less clear. The delayed influx of PI molecules observed in the delayed signature may be due to the generation of membrane defects that are too large to reseal but, initially too small to facilitate the influx of relatively large quantities of PI. We [18] and others [40] have previously observed this delayed molecular influx and have speculated that this signature is indicative of irreversible membrane poration, but with an initial phase of fewer and/or smaller electropores that are too numerous and/or too large for the cell to promptly repair the membrane. This speculation is supported by other studies where a critical electropore diameter has been explored. This critical electropore diameter, beyond which electropores expand rather than close, is thought to be roughly 20 nm [18], [45], [46]. Additionally, the delayed and rapid influx of PI observed in delayed IEP cells could be the result of initial membrane disruption added to downstream effects, including cell swelling due to osmotic imbalances across the membrane.

### Influence of Cationic Peptide Exposure on Membrane Disruption

**Figure 8:**
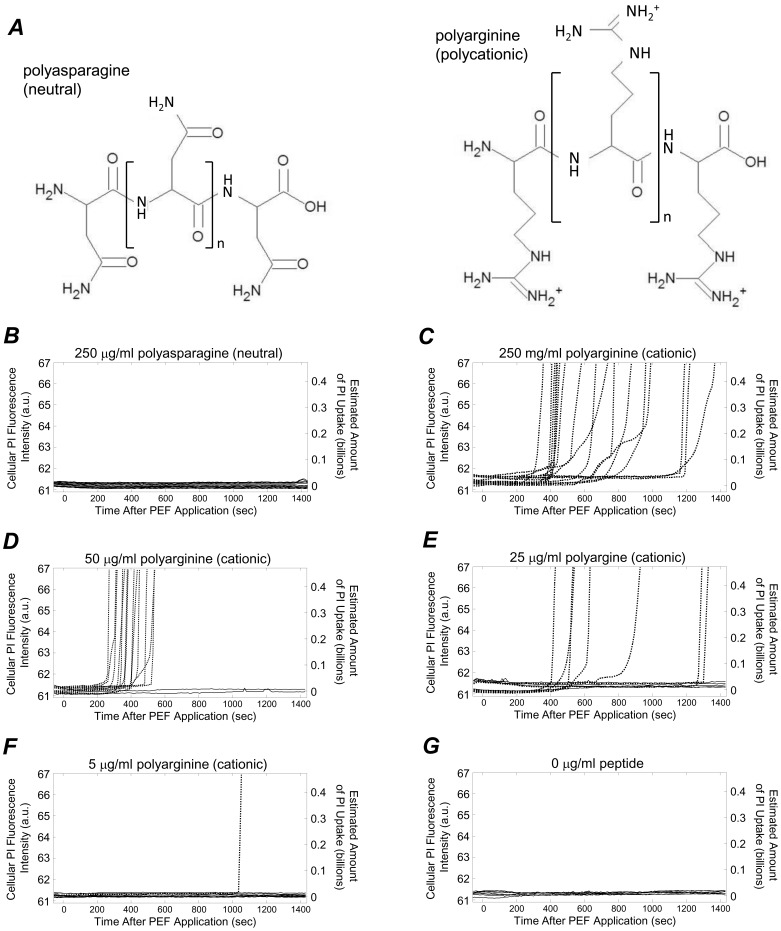
Cationic peptide exposure alone influenced membrane permeability to PI. ***A***: Cells were exposed to a peptides composed of neutral residues at neutral pH (polyasparagine, at left) and peptides with cationic residues at neutral pH (polyarginine, at right). These two peptides were of the same molecular weight. When 250 μg/ml of neutral polyasparagine or cationic polyarginine was added to cell media at −60 seconds, cells either fluoresced at baseline levels (***B***) or internalized PI in delayed, accelerating, and high levels (***C***). ***D***–***G***: Reducing the cationic peptide concentration reduced the number of cells that internalized PI during the course of the experiment, and increased the delay time observed for abrupt, accelerated, high-level PI uptake. In ***B***–***G***, the number of cells examined was 15, 18, 16, 14, 11, and 22, respectively.

Peptide exposure alone has an effect on membrane integrity. We performed experiments where cells were exposed to the charge-neutral polyasparagine (Fig. 8, ***A***, at left) and the polycation polyarginine (at right) while PI uptake was monitored over time. When 250 μg/ml of neutral polyasparagine was added at time *t* = −60 s, only the NEP signature was observed (Fig. 8, ***B***). At this same concentration of the cationic polyarginine, all cells exhibited the delayed IEP signature (Fig. 8, ***C***). Generally, when the cationic peptide concentration was decreased, the percentage of cells that exhibited the delayed IEP signature decreased and the percentage of cells that exhibited the NEP signature increased (Fig. 8, ***D***–***F***). Exclusive exhibition of the NEP signature was observed at 0 μg/ml peptide (Fig. 8, ***G***). These results indicate that after addition to the cell medium, cationic peptides interact with cell membranes, eventually resulting in significant membrane disruption. This is consistent with other reports, where a mechanism of cationic peptide toxicity has been described as electrostatic interaction, disruption, and poration of the plasma membrane [47], [48]. Elsewhere, the cationic peptide perforin was shown to trigger the membrane repair response in HeLa and CHO-K1 cells [28]. Our experimental observations are consistent with the mechanistic descriptions of cationic peptide cytotoxicity provided by Kościuczuk *et al* [47] and Niu *et al* [48], observations from Palm-Apergi *et al* [28], and the scenario proposed in Fig. 1. If cationic peptides are added to cell media at a certain time (Fig. 1, ***B***), it takes some time for them to diffuse in the cell media. Additionally, because of the negatively charged phosphatidylserine (PS) lipids in the plasma membrane’s inner bilayer, cationic peptides will electrostatically collect about the membrane over time (Fig. 1, ***C***). This leads to a delay in peptide-mediated membrane disruption, which would lead to delayed PI internalization (Fig. 1, ***D***). While it is possible that cationic peptide exposure initiates biochemical apoptotic pathways, eventually resulting in membrane permeability, the timescales associated with these rapid increases in PI permeability (hundreds of seconds) are quicker than would be expected through apoptotic means.

**Figure 9:**
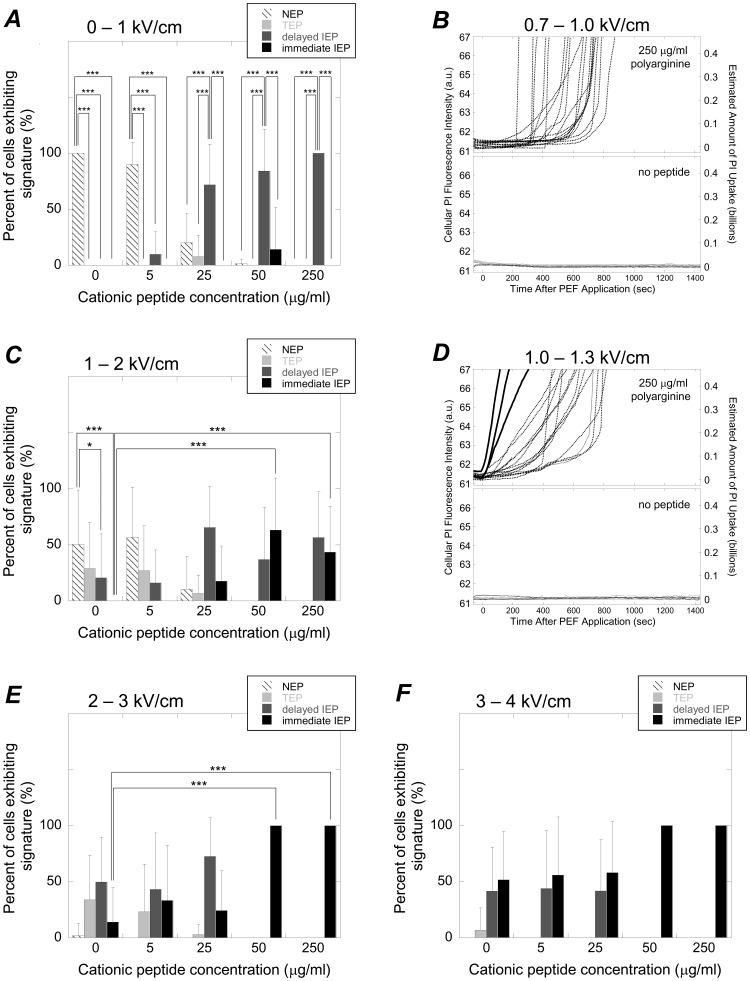
Cationic peptide exposure in concert with PEF application resulted in PI uptake signatures that were indicative of immediate and irreversible membrane disruption at lower PEF intensities. ***A***: Percent of cells exhibiting particular PI uptake signatures as a function of cationic peptide concentration from 0 to 1 kV/cm, highlighting the general absence of the immediate IEP signature. ***B***: Representative PI uptake curves at 0.7–1.0 kV/cm showing that peptide-exposed cells exhibited delayed, but not immediate IEP signatures (top) and that non-peptide-exposed cells exclusively exhibited the NEP signature (bottom). ***C***: Percent of cells exhibiting particular PI uptake signatures as a function of cationic peptide concentration from 1 to 2 kV/cm, highlighting the occurrence of the immediate IEP signature when using 50 to 250 μg/ml of cationic peptide. ***D***: Representative PI uptake curves at 1.0–1.3 kV/cm showing that peptide-exposed cells exhibited both IEP signatures (top) and that non-peptide-exposed cells exclusively exhibited the NEP signature (bottom). ***E*** and ***F***: Percent of cells exhibiting particular PI uptake signatures as a function of cationic peptide concentration at the indicated ranges in PEF intensities. In ***A***, ***C***, ***E***, and ***F***, data accounts for experiments that involved the use of 944 cells in 297 individual experiments. *, **, and *** represent statistically significant differences with *p* values of <0.05, 0.01, and 0.001, respectively (N = 5–29).

When administered prior to the time required for cationic peptides to disrupt membranes on their own, cationic peptide exposure could be used to reduce the PEF intensities required to induce immediate, irreversible membrane disruption. In control experiments where peptide was added and no PEF was provided, if PI uptake did occur, it was not manifest until at least 200 seconds into each experiment (Fig. 8). That is, the NEP and delayed IEP signatures dominated. Even in experiments where cationic peptide was used in concert with relatively low PEF intensities (0 to1 kV/cm), the NEP and delayed IEP signatures dominated at all cationic peptide concentrations tested (Fig. 9, ***A***). That is, at 0 and 5 μg/ml of polyarginine, the TEP signature was statistically more prevalent than all other signatures and at 25, 50, and 250 μg/ml, the delayed IEP signature was statistically most prevalent. Example PI uptake curves from experiments where 0.7–1.0 kV/cm PEFs were used with 250 and 0 μg/ml of polyarginine are provided in Fig. 9, ***B*** (top and bottom, respectively). Note that from 0 to 1 kV/cm, the signature associated with the most severe and immediate membrane disruption (i.e., the immediate IEP signature) was not observed in a statistically significant manner. However, from 1 to 2 kV/cm, the immediate IEP signature became more prevalent when using 50 and 250 μg/ml of cationic peptide (Fig. 9, ***C***, 50 and 250 μg/ml columns). In fact, the immediate IEP signature was not observed when no peptide was used but was observed at statistically higher percentages when using 50 and 250 μg/ml of polyarginine (Fig. 9, C, comparing the black bar in the 0 μg/ml column to the black bars in the 50 and 250 μg/ml columns). Also of note in this range in PEF intensities, is that the majority of cells exhibited the NEP signature when no peptide was used, indicating no membrane disruption. That is, from 1 to 2 kV/cm the NEP signature was observed roughly half the time and was statistically more prevalent than both the immediate and delayed IEP signatures (Fig. 9, ***C***, comparing the NEP bar to the delayed and immediate IEP bars in the 0 μg/ml column). This indicates that cell membranes can be predominantly undisrupted or severely disrupted at 1 to 2 kV/cm, depending on if they have been exposed to cationic peptide or not. More fine-tuned ranges of PEF intensities may improve this ability to selectively target cell membranes for disruption using cationic peptide. For instance, from 1.0–1.3 kV/cm, all cells that were not exposed to peptide did not internalize PI (Fig. 9, ***D***, bottom graph) but when exposed to 250 μg/ml of cationic peptide, some cells exhibited the immediate IEP signature (top graph). From 2 to 3 kV/cm, the immediate IEP signatures were exclusively exhibited when using 50 and 250 μg/ml of cationic peptide and were statistically more prevalent than when no peptide was used (Fig. 9, ***E***, comparing the immediate IEP bars in the 50 and 250 μg/ml columns to the immediate IEP bars in the 0 μg/ml column). From 3 to 4 kV/cm, the immediate IEP signature was not statistically more prevalent when using cationic peptide, likely due to the fact that this range of PEF intensities was adequate to generate a high degree of membrane disruption on its own.

**Figure 10:**
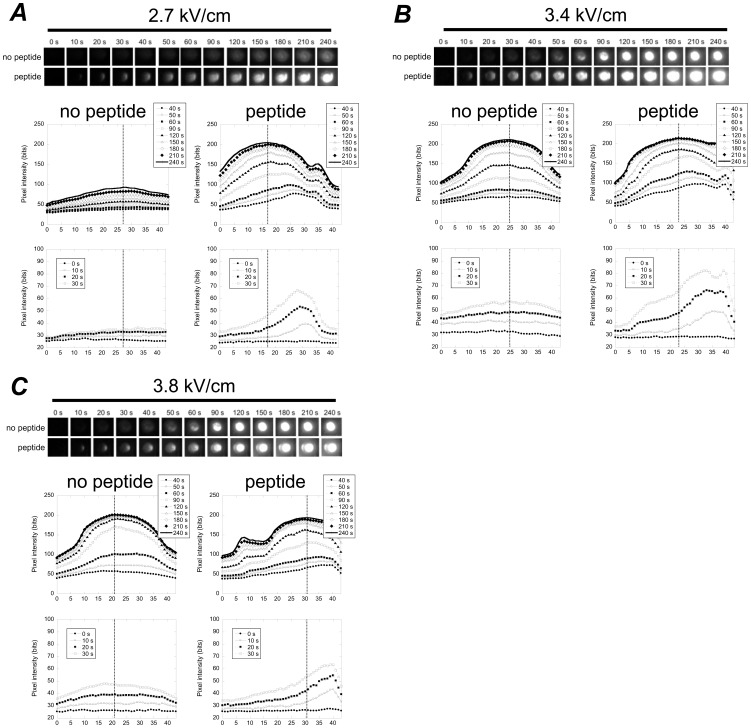
Experiments confirmed that cationic peptide exposure results in PI internalization, preferentially through the anode-facing membrane, particularly at time points immediately following PEF administration. Examples at 2.7/cm (***A***), 3.4 kV/cm (***B***), and 3.8 kV/cm (***C***), comparing real time fluorescence images during experiments revealed that cationic-peptide-exposed cells internalize PI asymmetrically (bottom fluorescence microscopy sequences, anode is at left) and non-peptide-exposed cells internalize PI symmetrically (top image sequences). This anodally preferenced PI uptake was most apparent at earlier time points (bottom graphs in each subfigure of the spatial distribution in PI fluorescence across the cellular axis). Top graphs in each subfigure show PI fluorescence axial distributions at later time points (40–240 seconds). The vertical line in each graph represents the point of maximum fluorescence at 240 seconds and is intended to estimate the cell center line. In each image and graph, the anode is at right.

The enhanced membrane disruption effects when cationic peptides were used in concert with PEFs were observed at the pole of the cell facing the anode, which is consistent with what we expected as illustrated in Fig. 2. Because cationic peptide location is subject to the directionality of the applied PEF, we expected that cationic peptides would only collect about the anodic pole during PEF application (Fig. 2, ***C***). Therefore, enhanced membrane disruption should be limited to the cell’s anodic pole (Fig. 2, ***D***) resulting in preferential PI internalization via the cell’s anodic pole (Fig. 2, ***E***). Indeed, this is what we observed during our experiments (Fig. 10). Anodic PI uptake has been reported elsewhere without the aid of cationic peptides [49]. Golizo *et al* [50] showed that this asymmetry was dependent on field strength and pulse duration with other contributing factors being cell population and size. Therefore, in order to use asymmetrical PI uptake as evidence to support claims that cationic peptide exposure is enhancing membrane disruption at the anode-facing membrane (as illustrated in Fig. 3), it is important to compare identical cell types, in situations where cell populations are similar and are exposed to identical pulse widths and intensities. Comparing identical cell types in cultures at 1 million cells per milliliter, when exposed to 2.7 kV/cm and 40 μs PEFs, cells that were not exposed to peptides internalized PI symmetrically (Fig. 10, ***A***, top row of fluorescence microscopy images). However, when exposed to cationic peptides under otherwise identical conditions, cells internalized PI with a strong bias of enhanced PI uptake on the anode side (Fig. 10, ***A***, bottom row of images). Cationic-peptide-exposed cells also internalized higher quantities of PI at earlier time points and internalized more PI by the end of the experiment (Fig. 10, ***A***, bottom four graphs comparing distribution in PI uptake over the axis of the cell parallel to the PEF). This indicates not only preferentially anodic PI uptake, but also more extensive membrane disruption due to peptide exposure. At higher PEF intensities, both peptide-free and cationic-peptide-exposed cells tended to both exhibit PI uptake signatures consistent with permanent electroporation, as expected. However, cationic-peptide-exposed cells internalized PI asymmetrically and internalized more PI at earlier points in time (Fig. 10, ***B***–***C***).

### Perspectives and Potential Directions

The use of polycationic materials has previously been explored to destroy unwanted cells types. Li *et al* [51] developed polycationic hydrogels that killed microbials through a proposed “anion sponge” where charged species disrupt membranes. Tew *et al* [52] and Gabriel *et al* [53] used polycationic polymers in solution to selectively disrupt bacterial membranes and kill bacteria. Such polycationic polymers tended to lose their bacteria-killing efficacy when immobilized (i.e., within a hydrogel) due to their inability to fully interact with the membrane of bacteria [51], [54], [55]. To compensate for this loss of efficacy, Sambhy *et al* [56] and Stratton *et al* [57] developed immobilized polycationic polymers with hydrophobic tails, aiding in their insertion and interaction with bacterial membranes. This approach also reportedly yielded high toxicity in mammalian cells [51], [56], [57]. Our findings also indicate that cationic materials, such as peptides, can be used to electrostatically interact with cell membranes, leading to irreversible membrane disruption. Use of cationic peptides, like the ones we have examined here, has several advantages for potential clinical adaptation, particularly with regard to their adaptability in molecular targeting strategies. For instance, peptides are biodegradable, relatively inexpensive and easy to produce, capable of being conjugated with cell-specific targeting molecules, and capable of integration within several drug delivery technologies. Additionally, as with other peptide-based therapies, their *in vivo* half-life may be extended through N- and C-termini blocking, cyclization, or 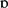-amino acid composition [58]. Also, cationic peptides require mere membrane co-localization–they need not be internalized to exert their cytotoxicity. Finally, as cell-specific targeting ligands are often also peptides (i.e., sequences of amino acids), cationic peptide domains may be conjugated with cell-specific targeting domains through relatively simple chemistry. This would potentially allow for targeted destruction of unwanted cells within complex tissues, which comprise inhomogeneous cell populations.

Our findings also demonstrate that exposure to cationic peptides can be used to lower the PEFs required to incur immediate and irreversible membrane disruption. Of particular note is that in the range of 1 to 2 kV/cm, cell membranes remained mostly un-disrupted (Fig. 9, ***C***, 0 μg/ml column). However, when 50 to 250 μg/ml of cationic peptide was used, a high proportion of cells experience immediate and irreversible membrane disruptions (Fig. 9, ***C***, 50 and 250 μg/ml columns). More specifically, at 1.0 to 1.3 kV/cm, peptide exposure caused a high degree of irreversible membrane disruption, while in the absence of peptides, all cell membranes remained un-disrupted (Fig. 9, ***D***). This indicates that at this range in PEF intensities, cell membranes can either be irreversibly disrupted or not disrupted, based on cationic peptide exposure. This ability to apply a PEF and only irreversibly damage membranes that have been co-localized with cationic peptide could be a powerful tool for molecularly targeting individual cells for destruction, particularly when coupled with ligand-receptor cell targeting technologies. A potential limitation to using cationic peptides in this manner, however, is their general toxicity. Targeting to specific cells may help, but it is also possible that tethering cationic peptides to larger structures may reduce accessibility of the peptide to interact with cell membranes, thereby reducing general cytotoxicity [51], [54], [55]. Furthermore, the charge density along the peptide may be tuned to minimize general cytotoxicity while still providing electroporation-enhancing effects. Additional studies would be required to determine if such strategies are fruitful and if reductions in membrane interactions would hinder the electroporative enhancing effects of cationic peptides. Finally, while this study uses cationic peptides in order to exploit their ability to electrostatically co-localize with the plasma membrane of cells, theoretically, co-localization of anionic peptides could also be used to enhance PEF-induced membrane disruptions. Though anionic peptides will not electrostatically collect about the membrane, they will be electrophoretically pulled to the cathodic membrane during PEF application, potentially enhancing membrane disruption on the cathode-facing pole of the cell. Use of anionic peptides may also have the added benefit of being less toxic and not prone to disrupting membranes in the absence of an externally applied PEF. That is, they may only contribute to membrane disruption during PEF application because they will only co-localize with the plasma membrane during pulse administration. A potential disadvantage, however, is that the ability for an anionic peptide to locally enhance the electrostatic potential across the membrane must fight against the cell’s resting potential. Additional studies would be required to examine their use in this respect.

Cationic peptide exposure may also prove to be a powerful tool in tissue engineering. Tissue engineering involves the development of biological constructs intended to repair, maintain, improve or replace dysfunctional or damaged tissues and organs [59]. Tissue engineering approaches typically require the use of biomaterials scaffolds upon which cells may mature and organize into a new or repaired tissue. One approach for obtaining such scaffolds has been to decellularize native tissues [60]. The native extracellular matrix (ECM) of a given tissue is a highly desirable tissue engineering scaffold in that (i) its components are generally conserved across individuals and even species, and thus are capable of implantation without rejection despite origin [61]–[64], (ii) it retains the complex three-dimensional (3D) architecture and mechanics required for proper tissue function, and (iii) it retains regionally specific cues for spatially organized cell adhesion [65]. Despite the fact that such scaffolds have been derived through decellularization of heart valves, blood vessels, skin, nerves, skeletal muscle, tendons, ligaments, small intestinal submucosa, urinary bladder, liver [60], heart, and kidneys [65], no single decellularization technique yields perfectly preserved ECMs. Therefore, multiple techniques must be employed to minimize ECM damage during decellularization, with careful consideration of how individual techniques affect specific ECM components [60]. Thus, additional methods of destroying cells without destroying ECM components would be greatly beneficial. Electroporative ablation may be a useful tool for decellularization as it has been shown to preferentially destroy membranes, leaving the ECM intact [24]. Cationic peptides, which can be perfused through a tissue’s endogenous vasculature, providing them access to that tissue/organ’s interior, could further enhance electroporative decellularization approaches.

## Conclusions

In conclusion, we have determined that cationic peptide exposure leads to significant and prolonged membrane disruption. When used in concert with externally applied PEFs, cationic peptide exposure results in high degrees of immediate membrane disruption at lower PEF intensities. This is most apparent when using PEFs ranging from 1 to 2 kV/cm, where in the absence of cationic peptide, PEF application did not result in accelerating uptake of PI immediately following PEF termination (i.e., the immediate IEP signature). However, with cationic peptide present, this PI uptake signature was widely observed, indicating a high degree of immediate membrane disruption. Additionally, when cationic peptides were present, PI delivery was preferentially anodic, as expected by theory, as the electric field direction results in collection of cationic peptide about the anodic membrane. While these studies suggest that cationic peptides may be useful in enhancing PEF-mediated membrane disruption and molecularly targeting cells for destruction, they do not directly address the mechanism for this enhancing effect. We thought in particular that cationic peptides would lower PEF intensities required for membrane disruption by influencing electrostatic potentials across the plasma membrane. Experiments involving direct measurement of transmembrane electrostatic potentials (e.g. patch clamp experiments or use of voltage-sensitive dies) will be required to fully understand the role that cationic peptides play in locally modifying electrostatic potentials across plasma membranes, providing mechanistic insight to the observations details in this report.
